# Parent and Family Functioning in Pediatric Inflammatory Bowel Disease

**DOI:** 10.3390/children7100188

**Published:** 2020-10-17

**Authors:** Grace Cushman, Sharon Shih, Bonney Reed

**Affiliations:** 1 Department of Psychology, University of Georgia, Athens, GA 30602, USA; gcushman@uga.edu; 2 Department of Psychology, Georgia State University, Atlanta, GA 30303, USA; sshih1@student.gsu.edu; 3 Emory + Children’s Pediatric Institute, Atlanta, GA 30322, USA

**Keywords:** family, gastroenterology, inflammatory bowel disease, parent, pediatric, psychogastroenterology

## Abstract

Although the impact of pediatric inflammatory bowel disease (IBD) extends beyond the patient to their parents and families, the focus of previous literature has largely been on investigating the patient’s medical and psychosocial functioning, with less consideration of the family system. Having a comprehensive understanding of parent and family functioning within the context of pediatric IBD is important given the role parents and family members have in the successful management of the disease and caring of the child. The current review paper aggregates the empirical research regarding parent and family functioning, including comparisons to normative samples, other illness groups, and how functioning relates to child psychosocial and health outcomes. Extant literature on parents and families in pediatric IBD has largely focused on the variables of parenting stress, parent psychosocial functioning, parent quality of life, and family functioning. Summary findings elucidate the complex relationships between parents, families, and children affected by IBD and highlight the importance of assessing parent and family functioning within pediatric IBD. The current review also offers implications for clinical practice, notes the limitations of the present literature, and provides recommendations for future research.

## 1. Introduction

The impact of pediatric chronic disease can be substantial for youth, their families, and their caregivers. Disruptions to daily life and increased stressors associated with illness may be particularly burdensome within pediatric inflammatory bowel disease (IBD), which is a group of disorders (e.g., Crohn’s disease, ulcerative colitis) characterized by inflammation in the gastrointestinal tract and symptoms including abdominal pain, fever, fatigue, diarrhea, hematochezia, weight loss, and growth delays [1,2,3]. While youth and their families attempt to cope with these potentially painful, embarrassing, and unpredictable symptoms, the side effects (e.g., bloating, distention, acne, and rapid weight changes) associated with treatments (e.g., steroids, biologic agents, and surgery) may also afflict additional burden on youth and their families [4]. Considering the combination of physical and psychological burdens, it is not surprising that patients are at an increased risk of poor psychosocial outcomes [5]. The psychosocial functioning of youth with IBD has been reviewed previously [3,6,7], and the cumulative results from this literature can be utilized to direct efforts in clinical settings, to inform prevention and intervention programming, and as a theoretical foundation to stimulate new research ideas. However, little focus has been given to aggregating findings related to parent and family functioning in pediatric IBD, despite the importance of the family system with regards to child health and psychosocial outcomes [8].

Across pediatric chronic health conditions, there is a large body of literature demonstrating the “undeniable impact” of a child’s illness on parents and families, with results interestingly demonstrating both positive and negative impacts on family functioning and parent psychosocial functioning [9]. Changes to a child’s health status can cause disruptions to individual and family routines and lead to lower satisfaction, leaving some family members to feel as though their needs are being unmet [10]. Within the marital relationship, it has been documented that certain dimensions of the relationship tend to be strengthened (e.g., communication and trust) while other aspects are susceptible to deterioration (e.g., sexuality); however, a dearth of research examining marital outcomes prevents broad conclusions [11]. Parents of youth with chronic conditions report restrictions on family activities, personal struggles (e.g., feelings of hopelessness, exhaustion), giving up jobs or careers to care for a child, and having difficulty finding the time to care for other children in the home [12]. Parents’ poor emotional well-being can have considerable impacts on the child diagnosed as chronically ill, as difficulties with psychosocial functioning may impede parents’ abilities to supervise and manage disease-related tasks (e.g., medication adherence) [13,14]. Looking beyond health outcomes, higher general and illness-related parenting stress has been associated with a worse psychological adjustment across youth with chronic conditions [13].

Parents are integral to the successful management of pediatric IBD, where they support and facilitate children’s medication adherence, schedule appointments and labs, and establish relationships with physicians [15]. Mothers, in particular, seem to be central to these tasks and responsibilities. In one study, mothers were most likely to be the individual responsible for making sure that adolescents with IBD had taken their medications, more so than fathers or the adolescents themselves. Furthermore, 89% of family members in the same sample indicated that mothers were primarily responsible for having and ordering more medications [16]. Greater maternal responsibility for the medication regimen has also predicted better adherence in youth with IBD [17].

Parent and family functioning reference broad areas of assessment; hence, the measurement of more nuanced subdomains within these constructs offers a richer understanding of how the family system relates to pediatric IBD outcomes. In parents of children with IBD, the assessment of parent functioning has investigated symptoms of psychopathology (e.g., anxiety, depression) primarily using standard measures of adult functioning (e.g., Brief Symptom Inventory-18) [18] as well as stress and distress related to parenting responsibilities. Parenting stress, often described as the stress associated with caring for a child with an illness [14], encompasses the frequency and difficulty of completing illness-related tasks for the child with a medical condition. In the pediatric IBD literature, parenting stress is measured most commonly using the Pediatric Inventory for Parents (PIP) [14], which provides scores aligning with this frequency and difficulty framework. Cognitive appraisals (e.g., rumination) and illness uncertainty (i.e., perceptions and uncertainty related to IBD symptom activity) have also recently gained momentum in the assessment of parent functioning [19,20].

Family functioning refers to dynamics within the family environment, both broadly and on more nuanced levels. Within the current pediatric IBD literature, family functioning has been examined in relation to child health and psychosocial outcomes. The specific domains of family functioning measured include communication, problem-solving, behavior control (i.e., expression and maintenance of appropriate behaviors of family members), affective responsiveness (i.e., expressing appropriate emotional responses), roles within the family, affective involvement (i.e., interest in and regard for others), conflict, and family member responsibility/involvement in certain tasks [8,17,21]. Past research has relied on generic measures of family functioning (e.g., Family Assessment Device) [21] as well as disease-specific measures (e.g., IBD Family Responsibility Questionnaire) [22].

Despite the growing number of studies examining parent and family functioning within pediatric IBD, a comprehensive review of the literature has yet to be completed. Therefore, the goals of the current narrative review were to (1) characterize parent and family psychosocial functioning difficulties across the domains of parenting stress, parent psychosocial functioning, parent quality of life, and family functioning, (2) examine those parent and family functioning domains in relation to child psychosocial and health outcomes, and (3) suggest areas for future research relevant to pediatric IBD. Based on the available literature, the current review focuses on parent and family functioning in relation to the child outcomes of health-related quality of life (HRQOL), psychosocial functioning, and disease activity and disease-related variables and largely includes cross-sectional and correlational findings, limiting causal implications.

## 2. Parenting Stress

In pediatric IBD, illness-specific parenting stress has been examined in relation to both child and parent demographic variables. Specifically, older caregiver age and completion of a four-year college degree have been associated with a lower frequency of parenting stress, while marital status and family socioeconomic status have not demonstrated a relationship with parenting stress [23]. Parents also experience comparable levels of parenting stress regardless of child age and gender [23,24]. Similar to findings in the larger child chronic illness literature [13], parents in one IBD sample most commonly endorsed items within the emotional distress domain of parenting stress [24].

### 2.1. Comparison to Other Illness Groups

On the PIP, the frequency of and difficulty with parenting stress in pediatric IBD was significantly lower than rates found in pediatric cancer, obesity, and sickle cell disease [23]. In comparison to parents of children with bladder exstrophy, parents of pediatric IBD patients reported significantly less difficulty with illness-related parenting stress but did not differ on the frequency of experiencing stressors [23]. Similar levels of parenting stress were found between pediatric IBD and type 1 diabetes [23]. Guilfoyle and colleagues further compared how parents of children with IBD and cancer differed on subscales of the PIP and found that the frequency of parenting stress associated with the subscales of Role Function (i.e., impact on the caregiving role within the home) and Emotional Functioning (i.e., emotional distress) were significantly lower in the IBD sample [23]. With regards to the level of difficulty associated with parenting stress, parents of youth with IBD reported significantly lower levels on the Communication, Medical Care, Role Function, and Emotional Distress subscales. Interestingly, in a sample of pediatric Crohn’s patients only, difficulty with, but not frequency of, parenting stress was significantly lower than in a pediatric cancer sample [25]. These findings suggest that parenting stress in IBD is generally lower or comparable to most other pediatric chronic illnesses. Moreover, it appears that even when the frequency of illness-related parenting stress is similar, the associated difficulty is significantly lower.

### 2.2. Relation to Child Outcomes 

In addition to characterizing how parents of youth with IBD compare to parents of other illness groups, past research has examined associations between parenting stress and child psychosocial and health outcomes. These relationships have largely been investigated using cross-sectional methods with concurrent correlation and mediation models; however, a limited number of longitudinal studies have also presented more complex statistical models.

#### 2.2.1. Child HRQOL

Overall, the extant literature demonstrates that higher parenting stress and distress in parents of youth with IBD have consistently been associated with lower child HRQOL. Within correlation models, parents of youth with IBD and parents of youth with only Crohn’s disease who endorsed higher parenting stress and distress had children with lower HRQOL [25,26]. Higher parental stress has also emerged as a significant mediator when examining a sample of parents of youth with Crohn’s disease, in that parental stress due to the occurrence and the perceived difficulty of medical stressors partially mediated the association between higher clinical disease activity and lower child HRQOL [25]. Within a sample of youth with IBD, parental distress also mediated the relationship between a shorter duration since the child’s last disease flare and child HRQOL, in that a shorter time since the last flare was associated with higher parental distress, which in turn, was related to lower child HRQOL [26].

#### 2.2.2. Child Psychosocial Functioning

Investigation of parenting stress in relation to child psychosocial functioning has examined child internalizing symptoms (e.g., anxiety, depression, somatization), externalizing symptoms (e.g., aggression), and global psychological functioning. Results consistently demonstrate that higher parenting stress is associated with worse overall child psychosocial functioning in cross-sectional studies and, although preliminarily examined, in longitudinal designs as well [24,27,28]. However, findings vary slightly depending on whether child psychological symptoms are self-reported (i.e., adolescent-reported) or parent-reported. In one study examining parent reports of child internalizing and externalizing problems [27], higher parenting stress was associated with worse internalizing and externalizing problems in adolescents with IBD when adolescent functioning was examined as a continuous variable. When placed into categories based on normal (i.e., average), borderline (i.e., slightly higher than average), and clinically elevated (i.e., clinically meaningful) internalizing symptoms, parenting stress due to the frequency or difficulty of medical events was significantly higher in the borderline and clinically elevated groups than in the group with scores within the normal range [27]. However, no between-group differences were found between externalizing symptom elevation groups. Additionally, higher parenting stress was associated with worse adolescent-reported internalizing, but not externalizing problems [27], as well as with worse overall psychological functioning [24] in adolescents with IBD. To our knowledge, studies have yet to examine the relation between parenting stress and child psychosocial functioning using self-reports of younger children (i.e., those <13 years old).

Although few studies have explored the longitudinal implications of parenting stress on child psychosocial outcomes, one preliminary study found that higher parenting stress predicted child depressive symptoms six months later, even when accounting for disease severity [28].

#### 2.2.3. Child Disease-Related Variables

Disease severity, as well as other disease-related variables, have also been examined in relation to parenting stress. Given that studies largely examined this relation cross-sectionally and with correlations, conclusions about disease severity causing parenting stress or the reverse relation cannot be inferred. Notably, findings regarding disease severity have had largely mixed results. Numerous studies have found that among adolescents with IBD, higher parenting stress (i.e., frequency and difficulty) has been associated with greater physician-reported disease severity and that baseline parenting stress has also been related to higher disease activity six months later [24,28]. A similar pattern was found among samples using only those diagnosed with Crohn’s disease; findings demonstrate significant relations between parenting stress frequency [25,27] and difficulty [23,25,27] and higher disease activity and severity. However, an association between parenting stress and disease severity has not been replicated in other IBD samples [26] and in samples limited to those diagnosed with ulcerative colitis [27].

Although only recently examined in the literature, preliminary results regarding other disease-related variables demonstrated that worse parenting stress was associated with a shorter time since the last IBD flare [26], whereas illness duration was unrelated to parenting stress [24].

## 3. Parent Psychosocial Functioning

In general, a moderate percentage of parents of youth with IBD report general emotional distress and psychosocial dysfunction [26,29]. In a sample of mothers only, 51% were found to have a lifetime history of depression based on a structured diagnostic interview [29], although most initial depressive episodes preceded the onset of the child’s illness, so causal implications due to the IBD diagnosis cannot be inferred. Similar levels of clinically elevated distress (47%) were reported by parents in a different, more recent study [26].

### 3.1. Comparison to Norms

Interestingly, distinctions between maternal and paternal psychosocial functioning have been examined in prior research, which allows for a more in-depth analysis of prevalence compared to norms. Fairly consistently across studies, mothers of youth with IBD report higher levels of psychological distress symptoms in comparison to healthy controls and normative samples [30,31,32]. In a study conducted with Swedish families of children with IBD, the domains of somatization, interpersonal sensitivity (i.e., feeling self-conscious or uncomfortable in interpersonal situations), depression, and anxiety on the Symptom Checklist 90 (SCL-90) were significantly elevated when compared to mothers of children without IBD [30]. In a different sample, Swiss mothers reported significantly higher symptoms of dysthymia and social phobia, as well as a higher overall symptom score compared to population norms on the short-form of the SCL-90 [32]. In a study conducted in the Czech Republic, mothers of youth with IBD reported moderate levels of anxiety as measured by the Beck Anxiety Inventory and endorsed significantly more symptoms of anxiety than mothers of healthy children [31]. However, there was no significant difference in symptoms of depression between mothers in the illness and control group in this sample [31].

In contrast, the literature on how paternal psychosocial functioning in pediatric IBD differs from normative populations is mixed. In a Swedish sample, fathers in the illness group reported similar levels of psychosocial symptoms to that of fathers in the healthy control group [30]. Surprisingly, in a Swiss sample collected more recently, fathers of children with IBD reported fewer symptoms of depression, agoraphobia, and social phobia, as well as improved overall functioning compared to the normative population [32]. However, a different study of fathers of adolescents with IBD found contradicting results, in that fathers reported higher levels of depressive symptoms on the Beck Depressive Inventory-II than a control sample [31]. Despite this significant difference, on average, depressive symptoms fell in the minimal range. Within this same sample of fathers, paternal symptoms of anxiety were comparable between the illness and control group [31]. For studies that have assessed differences between mothers and fathers of children with IBD, fathers consistently reported better mental health functioning than mothers [30,32].

Taken together, mothers of youth with IBD tend to report more psychosocial difficulty than mothers of healthy youth. Although it is less clear how fathers compare due to mixed findings, there is evidence that paternal psychological functioning is typically within the broad normative range. Additionally, fathers of children with IBD seem to fare better than their maternal counterparts when it comes to psychopathological symptoms.

Other research has examined broader areas of functioning without distinguishing by gender. When measuring psychosocial distress via the Distress Thermometer for Parents, parents of youth with IBD reported levels of distress similar to healthy controls across domains (i.e., practical, social, emotional, physical, and cognitive problems), except for parenting problems [26]. The parenting problem domain was significantly higher in the IBD group, which is not surprising given that elevated items in that domain reflect parenting a child with a chronic illness (i.e., more frequent difficulty talking to a child about disease or consequences of disease). Parents also reported a more frequent difficulty coping with fear but fewer problems with finances. Interestingly, the IBD group in this sample reported less paid employment than controls, which may be a consequence of needing to care for an ill child. Despite this difference in employment status, parents still endorsed fewer problems with finances [26]. Lastly, both mothers and fathers of youth with IBD reported significantly lower levels of social support than controls [30].

### 3.2. Comparison to Other Illness Groups

The rates of psychological adjustment difficulties in parents of youth with IBD as compared to other illness populations vary but suggest elevated to commensurate levels of impairment. Compared to mothers of children with cancer and renal disease, mothers of children with IBD demonstrated a higher proportion of insecure adult attachment (45% vs. 76%), as measured by the Adult Attachment Interview [33]. In addition, IBD mothers with insecure attachment scored higher than mothers of children with cancer and renal disease on a measure of psychiatric symptoms, including endorsing more symptoms of major depression [33]. In contrast, when compared to mothers of youth with cystic fibrosis, mothers of pediatric IBD patients demonstrated a comparable lifetime-prevalence of depression [29]. Across both the IBD and cystic fibrosis groups, the majority of mothers experienced their first episode of depression prior to the child’s illness diagnosis. However, within mothers of children with IBD, depression was associated with other psychopathologies, life events, and family strains [29]. This suggests that the determinants of maternal depression may differ across these populations. No group differences were found for generalized anxiety, but a higher prevalence of Obsessive Compulsive Disorder was found in IBD mothers and a higher prevalence of panic attacks was found in cystic fibrosis mothers. Lastly, no group differences were found between individuals with Crohn’s disease and ulcerative colitis [29]. It is important to note that, while novel, many of these findings reflecting differences between illness groups are based on one or two studies and should therefore be considered preliminary.

### 3.3. Relation to Child Outcomes

The stress and pressure associated with caring for a child with a chronic illness have the potential to impact parents’ global mental health functioning [31], as well as their mood and cognitions about their child’s disease. Therefore, it is unsurprising that numerous studies have examined parent psychosocial functioning in relation to child outcomes.

#### 3.3.1. Child HRQOL

Across studies, published findings consistently demonstrate that higher parent depressive symptoms and greater rumination have been associated with lower child HRQOL [19,34,35]. Two studies examining adolescents with IBD demonstrated correlational findings between higher caregiver depression and lower child HRQOL and also explored the role of caregiver depression in moderation [34] and mediation [35] models. Specifically, caregiver depression moderated the relation between adolescent depression and disease-specific HRQOL, in that higher adolescent depression was related to lower HRQOL, but only among youth whose parents had high levels of depression [34]. It was suggested that parents struggling with higher depressive symptoms may have difficulty facilitating appropriate communication and coping techniques for their adolescent, leading to lower HRQOL [34]. In a mediation model, disease symptoms were associated with greater child internalizing symptoms, which in turn related to higher caregiver depressive symptoms and lower HRQOL [35]. Findings extended previous research suggesting disease symptoms related to lower child HRQOL by positing that parent depressive symptoms were a mechanism through which this relation occurs. Additionally, one published abstract indicated that higher parent rumination about their child’s pain was associated with lower child HRQOL [19].

#### 3.3.2. Child Psychosocial Functioning

As in healthy youth, parent psychosocial functioning has repeatedly been examined with regards to child psychopathology and behavior problems in youth with IBD. Although results largely indicate that worse parent psychosocial functioning is related to worse child psychosocial functioning, only a limited number of studies have directly examined these associations [20,32,35,36]. Thus far, findings indicate a significant relation between caregiver depressive symptoms and adolescent internalizing symptoms, in that higher parent depressive symptoms were associated with higher adolescent internalizing symptoms [35]. A separate study observed a significant, negative association between overall maternal mental health and child behavior problems [32]. In addition to mental health symptoms, domains related to more state-like and disease-related cognitions have also been investigated. Correlationally, maternal positive affect has demonstrated an inverse relation with adolescent depressive symptoms [36], whereas mediation models demonstrated that parent illness uncertainty was related to youth illness uncertainty and, in turn, to higher youth depressive symptoms [20].

#### 3.3.3. Child Disease-Related Variables

Although studies have examined a diverse set of disease-related constructs in relation to psychosocial functioning, results generally indicate that worse disease-related functioning tends to co-occur with worse parent functioning [20,32,36,37]. One study found a positive association between parent illness uncertainty and overall disease activity [20], whereas more specific domains such as higher IBD-related disability and more frequent bowel movements have also been related to lower maternal positive affect [36]. Additionally, two studies found that greater child pain behavior and a shorter time since the child’s diagnosis were associated with higher parent pain catastrophizing and worse maternal/paternal mental health difficulties, respectively [32,37].

## 4. Parent Quality of Life (QOL)

### 4.1. Comparison to Norms

With regards to QOL, differing findings have been presented in the literature. In one study, both mothers and fathers of children with IBD reported lower overall QOL compared to controls on the PedsQL Family Impact Module [31]. In contrast, when parents of youth with IBD rated their QOL on the RAND 36, they endorsed significantly higher levels of QOL in multiple domains (i.e., physical functioning, role functioning, energy/fatigue, and general health) and comparable levels in other domains (i.e., emotional well-being, social functioning, and pain) compared to the normative sample [38]. To our knowledge, parent QOL in IBD has not been examined in relation to other chronic illness populations.

### 4.2. Relation to Child Outcomes

Parent QOL has not been examined as frequently as parenting stress or psychosocial functioning with regards to child outcomes, but preliminary results generally indicate that lower parental QOL has been associated with more negative child health and psychosocial functioning [38,39].

#### 4.2.1. Child HRQOL

Within one study of youth with IBD, higher child-reported mental and physical HRQOL was associated with higher parent mental QOL [38]. Interestingly, parents in this sample rated their QOL as best within the social and physical domains and as worst within the general health and energy/fatigue domains [38].

#### 4.2.2. Child Psychosocial Functioning

Regarding youth’s psychosocial functioning, better youth-reported emotional and behavioral adjustment demonstrated significant, positive relations with maternal, paternal, and family QOL in one sample [39]. However, findings are limited to a single study within this domain.

#### 4.2.3. Child Disease-Related Variables

Results from the few published studies examining disease-related variables and parent QOL largely indicate a significant, negative association between the two constructs. Specifically, worse disease activity was related to lower parent mental and physical QOL [38], as well as worse overall maternal, paternal, and family QOL [39].

## 5. Family Functioning

Beyond parents, pediatric illness is known to be associated with differences in systemic family functioning. In previous studies, a proportion of families affected by pediatric IBD reported clinically impaired difficulties in overall family functioning (13.3–24%), problem-solving (7.5–11.7%), communication (18.8%), roles (17.5%), affective responsiveness (12.5%), affective involvement (15–32.1%), and behavior control (7.5%) [27,40,41].

### 5.1. Comparison to Norms

Few studies have examined family functioning in relation to norms and other illness groups. However, when compared to parents of healthy adolescents, parents of youth with IBD have reported similar levels of family functioning in all domains except for increased impairments in family communication [42].

### 5.2. Comparison to Other Illness Groups

Maternal reports of the impact of the child’s illness on family members did not differ between IBD, cancer, and renal disease [33]. Although the literature is limited, existing evidence suggests that families of children with IBD demonstrate typical levels of family functioning.

### 5.3. Relation to Child Outcomes

Children with IBD spend the majority of their time within a larger family system. Regardless of the quality of medical care or school environments, family functioning is bound to influence a child’s adjustment. As one would imagine, across various domains of family functioning, higher levels are associated with better child functioning in pediatric IBD samples.

#### 5.3.1. Child HRQOL

Across studies, better family functioning has been consistently associated with higher levels of child HRQOL [8,43]. Specifically, in comparing families with and without clinically elevated difficulties in general family functioning, problem solving, and communication, families with clinically elevated difficulties endorsed significantly lower child HRQOL (i.e., social functioning, general well-being) [8]. Caes and colleagues [43] examined differences between youth and parent perspectives on family functioning in relation to youth-reported HRQOL and pain. Regression analyses revealed that higher youth-reported family satisfaction significantly contributed to better HRQOL. However, the relationship between HRQOL and parent-reported family satisfaction and cohesion was mediated by youth-reported pain intensity, so that higher family satisfaction and cohesion was related to lower pain intensity and, in turn, was associated with higher HRQOL [43]. Therefore, although findings demonstrate a pattern of association between family functioning and child HRQOL, the exact nature of the relationship may differ based on reporter perspectives and disease-related variables.

#### 5.3.2. Child Psychosocial Functioning

Family functioning in pediatric IBD has been studied in relation to domains of child psychosocial functioning including depression and behavioral functioning. Parent-reported family general dysfunction has not been found to significantly correlate with symptoms of depression in adolescents with IBD [36,40]. However, when examining specific domains of family functioning, worse family affective involvement (i.e., degree of family interest and involvement with one another) was positively correlated with parent-reported adolescent depressive symptoms, while better family problem-solving was negatively correlated with self-reported adolescent depressive symptoms [41]. Additionally, family stress has demonstrated significant, positive associations with youth self-reported depressive symptoms [44]. Although a direct comparison cannot be made, these results suggest that relationships between family functioning and adolescent depressive symptoms likely depend on specific domains of functioning as opposed to more global measures. Moreover, externalizing problems, but not internalizing problems, have demonstrated a significant relationship with general family functioning [40]. Of note, we did not identify any studies investigating the relationship between family functioning and other commonly examined psychosocial variables in pediatric IBD, such as symptoms of anxiety.

#### 5.3.3. Disease-Related Variables

The existing literature on the relationship between family functioning and IBD disease-related variables has predominantly focused on medication adherence and disease severity. While parent-child conflict has been identified both qualitatively and quantitatively as a barrier to adherence [17,45], youth with IBD describe family support and good parent-child relationships as facilitators of medication adherence [45]. In addition, better family functioning, characterized by the establishment of appropriate rules and consequences for behavior, has been found to correlate with improved adherence [46]. With regards to disease activity, higher family HRQOL was significantly associated with decreased youth disease activity [39], and general family dysfunction demonstrated a positive, significant relationship to pain/fatigue and the frequency of bowel movements [36]. Reed–Knight and colleagues [44] examined IBD pain-specific factors in relation to family stress and found that greater family stress was positively related to youths’ pain-related expressions of distress and passive pain coping. Across studies, results demonstrate a consistent relationship between aspects of family functioning and a variety of IBD outcomes, characterized by better family functioning and improved disease functioning. Please see Supplementary Table S1 for a comprehensive review of the primary findings.

## 6. Discussion

When a child is diagnosed with a chronic illness such as IBD, the illness and its treatment necessarily become the primary focus. Under traditional medical models, the illness may be treated with relatively little consideration of the child’s wider environmental milieu. As this review highlights, however, complex relationships exist between parent and family functioning and child psychosocial and health outcomes. Failure to consider these relationships will provide an artificially narrow view of a child’s diagnosis with IBD and will reduce options for promoting optimal health outcomes. Furthermore, consideration of parent and family functioning is consistent with current initiatives to assess and treat patients’ psychological difficulties as part of comprehensive IBD care [47]. Parent and family functioning may have varying and changing influences on child psychosocial and health outcomes across the developmental spectrum. We know the most about associations between parent and family functioning and child outcomes in adolescents with IBD, as this is where the majority of research has been conducted. It is imperative to maintain a developmental perspective in pediatrics, however, and not assume that relationships demonstrated in adolescents necessarily apply similarly to younger and older patients. Truly comprehensive IBD care will include consideration of parent and family functioning within the child’s developmental and psychosocial context.

Several conclusions, along with corresponding clinical implications, can be drawn from our review of parent and family functioning and child psychosocial and health outcomes in pediatric IBD. Across the examined domains, there are more consistent direct associations between parent and family functioning and child psychosocial outcomes in pediatric IBD as opposed to disease outcomes. This makes intuitive sense given the biological and treatment factors contributing to disease outcomes as well as the demonstrated relationships between parent and family functioning and child psychosocial health beyond patients with IBD. Although direct relationships between parent and family functioning and child health outcomes are less substantiated, evidence of moderated and mediated effects suggest that more complex relationships may exist and warrant further exploration [25]. Second, it is likely that parent and family functioning prior to a child’s diagnosis with IBD are strong predictors of postdiagnosis functioning. For example, Burke and colleagues conducted diagnostic interviews with mothers of youth with IBD and found that 51% reported a history of depressive episodes [29]. However, the onset of depression preceded the child’s IBD diagnosis for the majority of mothers, suggesting that premorbid psychological functioning was at least partially responsible for the prevalence of parental psychopathology in the sample. Though logistically difficult to conduct, studies examining the role of parent and family functioning prior to a child’s diagnosis will be important in making conclusions about the relative impact of premorbid functioning compared to disease onset.

### 6.1. Clinical Implications

Given the associations between parent and family factors and patient outcomes, family-based psychosocial assessments are likely warranted in newly-diagnosed pediatric IBD patients. Psychologists working with patients would likely need to explain the relevance of a family-based assessment to the child’s overall health and functioning and could include domains of parent psychopathology as well as general family functioning. When introducing a family-based assessment, a psychologist can describe the findings from this review, highlighting that past research has demonstrated associations between parent and family functioning and child outcomes. In order to most comprehensively care for their child with IBD, family-based psychosocial assessments offer the opportunity to identify areas of difficulty that may have implications for the child’s overall health and well-being [48]. For those families reporting distress, appropriate treatment recommendations may include individual or family therapy as well as parent groups focusing on self-care and parenting a child with chronic illness, where available. Interventions aimed at family functioning may choose to focus on those domains that have been most consistently related to differential outcomes in pediatric IBD, including family problem-solving and communication [8].

### 6.2. Limitations

In conducting this narrative review, we encountered limitations in the existing body of research on parent and family functioning in pediatric IBD that constrain conclusions and clinical implications but offer opportunities for future research. Arguably most importantly, we frequently relied on a single or few studies within each domain reviewed. Consequently, one must be careful about drawing conclusions about consistent relationships between parent and family functioning and child outcomes before findings are replicated. This is particularly notable when considering findings comparing parent and family functioning between IBD and other chronic illness groups, as these groups differ across many characteristics that were not controlled for and replication studies are lacking. In addition, many of the available studies are now older and may not capture patients’ current functioning, especially with medical progress in the treatment of IBD. Within the available family research, there are very limited data available on siblings, and the majority of past work has been conducted with mothers. In work that has included fathers, they tend to report more adaptive functioning compared to their family counterparts, suggesting that fathers offer a unique perspective and need to be included in future research to garner a comprehensive understanding of family functioning. With regard to the patient participants, less is known about parent and family functioning in patients diagnosed at a young age, especially with very early onset IBD. As youth are most commonly diagnosed in early adolescence, the majority of research available was on older children and adolescents, limiting evaluations of differences between adolescents and younger children. Although multiple domains of family functioning have been evaluated, less is known about the impacts of pediatric IBD on the marital relationship and how the marital relationship in turn relates to child outcomes. The majority of available studies were conducted in the United States with predominantly Caucasian samples, limiting conclusions regarding potential differences based on nationality, culture, and race. Finally, given the novelty of the current review in aggregating available data on parent and family functioning, a systematic review and meta-analysis were unable to be conducted. We hope the current review provides the theoretical foundation needed to identify strict inclusion and exclusion criteria and specific aims for such future reviews.

### 6.3. Future Research Directions

The future of biopsychosocially based research in pediatric IBD seems bright, with increasing interest in patients’ complete illness experience beyond diagnosis and medical management. In Table 1, we summarize limitations of existing research, as well as suggestions for future research. We look forward to increasing focus on the potentially protective roles that parent and family functioning can play in child outcomes. Initial research in the field has understandably focused on negative outcomes (e.g., depression, poor HRQOL) and how parent and family functioning are associated. But it will be equally important to identify parent and family factors associated with optimal functioning and even thriving within the context of pediatric IBD. Additional parent and family factors should also be investigated. For example, parenting style research is a burgeoning area of inquiry, with interest into how patterns of parents’ attitudes, strategies, and typical ways of relating to their children are associated with child outcomes in pediatric IBD [31]. Longitudinal studies across the disease course are also needed to identify if critical periods exist for the role of parent and family functioning, as well as changes across development and time since diagnosis. Longitudinal examinations will also allow for more comprehensive models of parent and family functioning that examine the interrelationships between factors (e.g., parent QOL and parent psychopathology). There is likely to be considerable overlap between these constructs of interest, and only by assessing and modeling them simultaneously will we begin to understand core areas of functioning. Increasing focus on cross-cultural samples and ethnically and racially diverse samples is needed to inform culturally relevant assessments and interventions. Furthermore, as previous literature has demonstrated disparities between reporters [27,43], the examination of both child and parent reports can provide a more comprehensive understanding of parent and family functioning. Once adequate data are available, we look forward to meta-analytic studies to synthesize past research and capitalize on multiple studies in order to form conclusions.

In conclusion, the extant literature on parent and family functioning in pediatric IBD reflects an area of inquiry that is in its nascent stages. Our findings indicate that there are important associations between the family system and a child with IBD but that the nuance of how these factors mutually affect each other is less clear. This presents a robust area for future research that builds upon our understanding of the complex relationship between parents, families, and children with IBD.

## Figures and Tables

**Table 1:** Summary of limitations and future directions for research on parent and family functioning in pediatric IBD.

Limitations	Future Directions
Current literature base lacks replication, is primarily cross-sectional, and many studies are now older	Need for longitudinal investigations Need for greater diversity within samples across races, ethnicities, nationalities, and patient ages
Current research primarily relies on maternal report	Increased inclusion of paternal-report and siblings
Limited domains of family functioning have been evaluated	Examination of marital relationship post-diagnosis
Within parent functioning, focus has been on parenting stress, psychosocial functioning, and QOL	Additional areas of study may include parent personality domains, parenting style, parent coping and social support, and relationships between parents and healthcare providers
To date, focus has been on distress-related outcomes	Assessment of protective factors and positive psychological constructs
Individual studies with single-site data collection	Meta-analytic investigations along with multi-site participant enrollment
	Developmental changes in parent and family functioning across time since diagnosis and child age
	Multi-reporter assessment
	Comparison of HRQOL in parents of youth with IBD to other illness groups
	Developing more comprehensive models of parent and family functioning that account for interrelationships between constructs

IBD = Inflammatory bowel disease; QOL = quality of life; HRQOL = health-related quality of life.

## Supplementary Materials

The following are available online at https://www.mdpi.com/2227-9067/7/10/188/s1, Table S1: Summary of Results.
