# Supplementary material for: Parent and Family Functioning in Pediatric Inflammatory Bowel Disease
Source: Children (Basel). 2020 Oct 17;7(10):188. doi: 10.3390/children7100188 (PMC7603067; doi:10.3390/children7100188)
Supplement: Supplementary file 1 [file children-07-00188-s001.pdf]

First Author (Year)	Parent/Family variable(s) examined	Child variable(s) examined	Sample Characteristics	Comparison Group(s) (if applicable)	Measures Used	Primary Results
Ahola-Kohut (2018)	Pain Catastrophizing	HRQOL<br>Pain Intensity<br>Disease Activity	Canada<br>*N*=71<br>Youth (M_{age} = 15.21±1.49) & Caregivers<br><br>CD *n*=45<br>UC *n*=24<br>IBD-U = 2	N/A	Not Reported	Parent pain catastrophizing (rumination about child pain) was significantly associated with lower youth HRQOL
Baudino (2018)	Illness Uncertainty	Depression,<br>Disease Activity	United States<br>*N*=85<br>Youth (M_{age} = 15.75±1.51; 74.1% White) & Caregivers (M_{age} = 43.9±6.7)<br><br>CD *n*=50<br>UC *n*=35	N/A	CDI-2,<br>Child Uncertainty in Illness Scale,<br>Parent Perceptions of Uncertainty Scale,<br>PGA	Parent illness uncertainty has a significant influence on youth illness uncertainty appraisals, which in turn relates to elevated child depressive symptoms<br>Parent illness uncertainty related to worse youth disease activity
Burke (1994)	Psychosocial Functioning<br>Family Functioning	N/A	United States<br>*N*=72<br>Mothers (M_{age} = 39.5±5.12) of youth with IBD (M_{age} = 12.22±2.71)<br><br>CD *n*=49<br>UC *n*=23	Cystic fibrosis (CF)	A-SADS-L,<br>FILE,<br>FRI	51% of IBD mothers reported a history of depression compared to 41% of CF mothers<br>First episode of maternal depression preceded IBD diagnosis<br>Maternal depression associated with other psychopathology, life events, and family strains<br>Higher prevalence of Obsessive Compulsive Disorder in IBD mothers<br>No significant differences in parent psychosocial functioning between CD and UC
Caes (2019)	Family Functioning	HRQOL<br>Pain Intensity	Canada<br>*N*=60<br>Youth (M_{age} = 13.37±2.54) & Parents (M_{age} = 45.30±6.13)<br><br>CD *n*=41<br>UC *n*=17<br>IBD-U *n*=2	N/A	Faces Pain Scale-Revised,<br>FACES-IV,<br>IMPACT-III,<br>PGA	Parents reported significantly higher levels of family cohesion and communication, but significantly lower satisfaction as compared to youth<br>Higher youth-reported family satisfaction and lower pain intensity related to better youth HRQOL<br>Higher parent-reported family cohesion and satisfaction indirectly related to youth HRQOL through lower pain intensity

Diederen (2018)	Parental Distress	HRQOL<br>Disease Activity	Netherlands<br>*N*=87<br>Youth (Median age = 15.2, IQR = 13.4-16.5)<br>Parents (Median age = 45.8, IQR = 42.6-50.6)<br><br>CD *n*=58<br>UC *n*=29	Healthy Controls	Distress Thermometer for Parents,<br>PedsQL,<br>PUCAI,<br>shPCDAI	47% of IBD parents reported clinically elevated distress, though this was not significantly different from parents of healthy controls<br>Similar levels of parental distress between IBD parents and healthy control parents, but IBD parents reported more frequent parenting problems<br>Increased parental distress associated with lower child HRQOL and shorter time since last disease flare<br>Parental distress mediated the relationship between shorter duration since child's last disease flare and child HRQOL, such that shorter time since the last flare was associated with higher parental distress, which in turn was related to lower child HRQOL
Engstrom (1991)	Parental Distress<br>Social Support	N/A	Sweden<br>*N*=20<br>Youth (M_{age} = 16.5)<br>Mothers (M_{age} = 42.7)<br>Fathers (M_{age} = 44.2)<br><br>CD *n*=9<br>UC *n*=11	Healthy Controls	ISSI,<br>SCL-90	IBD mothers report higher levels of parental distress, somatization, interpersonal sensitivity, depression, and anxiety than healthy control mothers<br>IBD fathers report similar levels of parental distress to healthy control fathers<br>IBD mothers and fathers report lower levels of social support compared to healthy control mothers and fathers
Gray (2013)	Parenting Stress<br>Family Functioning	Emotional/Behavioral Functioning<br>Disease Severity	United States<br>*N*=130<br>Youth (M_{age} = 15.64±1.36; 81.5% White) & Caregivers<br><br>CD *n*=100<br>UC *n*=30	Normative Sample	CBCL,<br>YSR,<br>FAD,<br>LCAI,<br>PIP,<br>shPCDAI,	Higher parenting stress is associated with higher levels of parent-report internalizing and externalizing symptoms.<br>Parents of youth with borderline or clinical levels of internalizing symptoms had significantly more parenting stress than parents of youth with normal levels.<br>Higher parenting stress associated with higher levels of youth-report internalizing but not externalizing symptoms<br>In CD patients, higher parenting stress was associated with higher levels of disease severity. No relationship found in UC.<br>Poorer family functioning associated with increased parenting stress
Gray (2015)	Parenting Stress	HRQOL<br>Disease Activity	United States<br>*N*=99<br>Youth (M_{age} = 15.16±1.37; 79.8% White) & Caregivers<br><br>CD *n*=99	Pediatric cancer	IMPACT-III,<br>PIP,<br>shPCDAI	Difficulty with, but not frequency of, parenting stress was significantly lower in Crohn's disease than in pediatric cancer<br>Higher parenting stress associated with lower youth HRQOL<br>Higher disease activity and lower youth HRQOL mediated by parenting stress due to the frequency and difficulty of medical stressors
Greenley (2009)	QoL	Disease Activity<br>QoL	United States<br>*N*=49<br>Youth (M_{age} = 14.96±2.25; 90% White) &	Normative Sample	CHQ-CF 87,<br>Myren modification of the Harvey Bradshaw Simple Index,<br>RAND 36	Parents of youth with IBD reported higher/better QoL in 5 of 8 domains when compared to normative group: physical functioning, role limitations due to physical health, role limitations due to emotional problems, energy/fatigue, and general health<br>Greater youth disease activity was associated with lower parent QoL in mental health and physical health domains

			Parents (M_{age} = 42.86±6.33; 92% White)			Higher youth mental and physical OQL was associated with higher parent QoL in the mental health domain<br>Parent QoL rated as best in social and physical domains and worst within the general health and energy/fatigue domains
Guilfoyle (2012)	Parenting stress	Disease Activity	United States<br>*N*=62<br>Youth (M_{age} = 15.4±1.4; 88.7% White)<br>Caregiver (M_{age} = 45.7±5.6)<br><br>CD *n*=49<br>UC *n*=13	Pediatric cancer, obesity, sickle cell disease, bladder exstrophy, type 1 diabetes	LCAI,<br>PCDAI,<br>PIP,	Lower frequency of parenting stress associated with older parent age and 4-year college degree<br>Marital status and family socioeconomic status not associated with parenting stress<br>Lower parenting stress frequency in IBD than cancer, obesity, and sickle cell disease<br>Lower parenting stress difficulty in IBD than cancer, obesity, sickle cell disease, and bladder exstrophy<br>Similar levels of parenting stress frequency and difficulty in IBD compared to Type 1 diabetes<br>Compared to cancer, frequency of parenting stress associated with the subscales of Role Function and Emotional Functioning were lower in IBD<br>Compared to cancer, difficulty of parenting stress associated with the subscales of Communication, Medical Care, Role Function, and Emotional Distress was lower in IBD<br>Crohn's disease activity associated with parenting stress difficulty
Guilfoyle (2014)	Parenting Stress	Depression<br>Disease Activity	United States<br>*N*=93<br>Youth (M_{age} = 15.5±1.4; 91.7% White) &<br>Parents (M_{age} = 45.7±5.6)<br><br>CD *n*=74<br>UC *n*=10<br>Not reported *n*=9	N/A	CDI,<br>LCAI,<br>PCDAI,<br>PIP	Parenting stress at baseline accounted for significant variance in youth depressive symptoms at 6 months, controlling for disease severity<br>At baseline, parenting stress frequency and difficulty associated with disease symptoms.<br>At 6 months follow-up, only parenting stress frequency associated with disease symptoms.
Herzer (2011)	Family Functioning	Disease Severity<br>HRQOL	United States<br>*N*=62<br>Youth (M_{age} = 15.47±1.41; 88.7% White) & Caregivers<br><br>CD *n*=49<br>UC *n*=13	N/A	FAD,<br>IMPACT-III,<br>LCAI,<br>PCDAI	Parent report of clinically elevated difficulties in some aspect of family functioning ranged from 7.5%-25% of the sample<br>Greater familial difficulties with problem solving and communication, and greater difficulties in general family functioning associated with lower child HRQOL after controlling for disease severity
Hommel (2011)	Family Barriers to Treatment Adherence	Treatment Adherence	United States<br>*N*=16	N/A	LCAI,<br>PCDAI,	Qualitative association between parent-child conflict about taking medications and poor treatment adherence

			Youth (M_{age} = 15.75±1.08; 94% White)<br>*N*=12 Mothers (M_{age} = 46.44±5.32)<br>*N*=4 Fathers (M_{age} = 48.81±7.49)<br><br>CD *n*=15<br>UC *n*=1		Treatment adherence interview	Family support, good parent-child relationship, and parental monitoring reported as facilitators to treatment adherence<br>Resolution of familial conflict by discussion rather than fighting enhanced adherence outcomes<br>Discussion about importance of transitioning allocation of responsibility to children from parents, but parents expressed uncertainty about when and how
Jelenova (2016)	Parenting Style<br>Anxiety<br>Depression<br>QoL	QoL<br>Anxiety<br>Depression	Czech Republic<br>*N*=27<br>Youth (M_{age} = 15.1±1.20) & Parents<br><br>CD *n*=17<br>UC *n*=10	Healthy Controls	ADOR,<br>BAI,<br>BDI-II,<br>CDI,<br>KidScreen-10,<br>PedsQL Family Impact Module,<br>SAD	IBD mothers reported moderate levels of anxiety and significantly more anxiety than healthy control mothers. No difference in depression symptoms.<br>IBD fathers reported higher levels of depression than healthy control fathers. No difference in anxiety symptoms.<br>Lower QoL in IBD parents versus healthy control parents<br>No difference in parenting style between IBD and healthy control parents, except for fathers' positive parenting higher among controls.<br>Parenting style among IBD parents significantly related to child QoL, anxiety, and depression.
Kunz (2011)	Family QoL<br>Parent QoL	Disease Activity<br>Psychosocial Functioning	United States<br>*N*=95<br>Youth (M_{age} = 15.07±2.16; 96% White)<br>*N*=92 Mothers (M_{age} = 44.44±4.98)<br>*N*=43 Fathers (M_{age} = 45.54±5.81)<br><br>CD *n*=80<br>UC *n*=12<br>IC *n*=3	N/A	Pediatric Symptoms Checklist,<br>PedsQL Family Impact Module,<br>PGA	Youth-reported emotional and behavioral adjustment demonstrated significant, positive relations with maternal, paternal, and family QOL<br>Worse disease activity associated with worse overall maternal, paternal, and family QoL<br>No significant differences in maternal and paternal reports of parent of family QOL.
Langer (2014)	Pain Catastrophizing<br>Parent Responses to Pain	Pain Behavior	United States<br>*N*=184<br>Youth (M_{age} = 13.72±2.72; 88% White) &<br>Parents (M_{age} = 44.37±6.85; 93% White)<br><br>CD *n*=126	N/A	Adult Responses to Children's Symptoms,<br>Faces Pain Scale Revised,<br>Pain Behavior Checklist,<br>Pain Catastrophizing Scale – Parent	Parent pain catastrophizing was positively associated with greater child pain behavior.<br>Parent pain catastrophizing mediated the relationship between parent-reported child pain behavior and parental protective responses

			UC n=58			
Loreaux (2015)	Depression	Depression<br>HRQOL	United States<br>N=86<br>Youth (M_{age} = 14.10±1.93; 91.8% White)<br><br>CD n=65<br>UC n=18<br>IC n=3		BASC-2,<br>BSI,<br>IMPACT-III<br>Partial Harvey-Bradshaw Index<br>PUCAI	Caregiver depression moderated the relation between youth depression and HRQOL, after controlling for disease activity, only among among youth whose parents reported high levels of depression
Mackner (2005)	Family Functioning	Medication Adherence	United States<br>N=50<br>Youth (M_{age} = 14.69±1.92; 90% White) & Parents<br><br>CD n=38<br>UC n=4<br>IBD-U n=8	N/A	Adherence interview,<br>FAD	Better family functioning, especially establishing rules and consequences for behavior, was associated with higher adherence
Mackner (2006)	Family Functioning	N/A	United States<br>N=50<br>Youth (M_{age} = 14.39; 87% White) & Parents	Healthy Controls	FAD	Similar levels of family functioning between youth with IBD and healthy controls, except for increased impairments in family communication in IBD
Odell (2011)	Parenting Stress<br>Family Functioning	Behavioral Problems<br>Depression<br>Disease Severity	United States<br>N=45<br>Youth (M_{age} = 15.41±1.32; 89% White) & Parents<br><br>CD n=36<br>UC n=9	N/A	CBCL,<br>CDI,<br>FAD,<br>LCAI,<br>PIP,<br>PUCAI.<br>YSR	Parent-reported family dysfunction was not significantly related to youth depressive symptoms or disease severity.<br>Child externalizing problems, but not internalizing problems, significantly associated with family dysfunction<br>Parenting stress not significantly related to family functioning
Plevinsky (2018)	Parenting stress<br>Psychosocial Functioning	Psychosocial Functioning<br>Disease Activity	United States<br>N=51<br>Youth (M_{age} = 17.92±0.94; 86.3% White) & Parents<br><br>CD n=37<br>UC n=13<br>IC n=1	Younger IBD sample (N=48; M_{age} = 14.89±1.15)	BSI,<br>PGA,<br>PIP	Higher parenting stress frequency and difficulty associated with worse youth and parent psychosocial functioning, and worse disease activity<br>Child age and gender not related to parenting stress<br>Items in emotional distress domain most commonly endorsed<br>Parenting stress unrelated to illness duration<br>Baseline parenting stress related to higher disease activity 6 months later

Reed-Knight (2011)	Family Responsibility, Sources of parent-child conflict	Medication Adherence	United States<br>N=90<br>Youth (M_{age} = 14.72±2.24; 78% White) & Caregivers<br><br>CD n=67<br>UC n=23	N/A	IBD Family Responsibility Questionnaire,<br>Issues Checklist,<br>Medical Adherence Measure	Less perceived conflict between parent and child and greater maternal involvement in IBD management was associated with better medication adherence<br>No associations were found between adolescent-reported adherence and parent-child conflict or maternal involvement.
Reed-Knight (2016)	Depression	Disease Activity, HRQOL, Internalizing Symptoms	United States<br>N=83<br>Youth (M_{age} = 14.77±2.29; 78.3% White) & Caregivers<br><br>CD n=63<br>UC n=20	N/A	BASC-2,<br>IMPACT-III,<br>PCDAI,<br>PUCAI,<br>SCL-90 Revised	Greater disease activity was associated with greater child internalizing symptoms, which in turn related to higher caregiver depressive symptoms and lower caregiver HRQOL
Reed-Knight (2018)	Family Stress	Depression, Pain Coping, Pain Distress	United States<br>N=183<br>Youth (M_{age} = 13.75±2.70; 88% White) &<br>Parents (M_{age} = 44.38±6.87; 92.9%)<br><br>CD n=125<br>UC n=58	N/A	CDI,<br>FILE,<br>Pain Behavior Checklist,<br>Pain Response Inventory	Parents most commonly reported family stressors related to financial strain regarding medical expenses, food, clothing, energy, and home care.<br>Greater family stress positively related to children's pain-related expressions of distress and passive coping<br>Family stress positively related to youth self-reported depressive symptoms
Schuman (2013)	Family Functioning	Depression, Disease Severity	United States<br>N=122<br>Youth (M_{age} = 15.7±1.3; 86.7% White) & Parents<br><br>CD n=96<br>UC n=26	N/A	CBCL,<br>CDI,<br>FAD,<br>LCAI,<br>PCDAI	Approximately 12% and 32% of parents reported clinically significant difficulties with family problem-solving and affective involvement, respectively.<br>The specific family functioning domain of family affective involvement was positively associated with parent-reported youth depressive symptoms.<br>The specific family functioning domain of family problem-solving was negatively associated with self-reported youth depressive symptoms.<br>Neither family functioning variable moderated the relationship between disease activity and depressive symptoms.
Szajnberg (2011)	Attachment Psychosocial Functioning	Disease Severity	United States<br>N=21<br>Youth (M_{age} = 12.8) & Mothers<br><br>CD n=16<br>UC n=5	Pediatric cancer and renal disease	Adult Attachment Interview,<br>Clinician's Objective Burden of Illness Index,<br>Impact on the Family Scale,	IBD mothers had a higher proportion of insecure adult attachment as compared to pediatric cancer and renal disease mothers.<br>IBD mothers with insecure attachment scored higher on measures of psychiatric symptoms, including depression<br>No differences found between groups on the impact of the child's illness on family members

					Million Clinical Multi-Axial Inventory-II	
Tojek (2002)	Family Functioning<br>Physical Signs and Symptoms<br>Positive and Negative Affect	Bowel Movement Frequency, Depression, Functional Disability, Pain and Fatigue	United States<br>*N*=62<br>Youth (M_{age} = 15.05) & Mothers<br><br>CD *n*=35<br>UC *n=26*	N/A	CDI,<br>FAD,<br>Functional Disability Index,<br>Pennebaker Inventory of Limbic Languidness,<br>Positive and Negative Affect Schedule	Family dysfunction positively correlates with youth bowel movement frequency and pain/fatigue<br>Family dysfunction was not correlated with youth depression<br>Mother's positive affect was negatively correlated with youth depression, functional disability, and bowel movement frequency
Werner (2015)	Psychosocial Functioning	Behavior Problems<br>Time since diagnosis	Switzerland<br>*N*=125<br>Youth (M_{age} = 13.3±2.8)<br>*N*=125 Mothers (M_{age} = 43.7±4.8)<br>*N*=106 Fathers (M_{age} = 46.4±5.5)<br><br>CD *n*=70<br>UC/IC *n*=55	Normative Sample	Strengths and Difficulties Questionnaire,<br>Symptom Checklist 27	IBD mothers reported significantly more symptoms of dysthymia, social phobia, and overall symptom score than the normative sample<br>IBD fathers reported fewer symptoms of depression, agoraphobia, social phobia, and better overall functioning compared to normative sample<br>Poorer mental health in both parents was associated with shorter time since child's IBD diagnosis<br>Higher levels of child behavior problems associated with poorer maternal mental health

A-SADS-L = Adult Schedule for Affective Disorders and Schizophrenia, Lifetime Version; BAI = Beck Anxiety Inventory; BASC-2 = Behavior Assessment Scale for Children, second edition; BDI-II = Beck Depression Inventory, second version; BSI = Brief Symptom Inventory; CAS = Children Assessment Schedule; CBCL = Child Behavior Checklist; CD = Crohn's disease; CDI = Children's Depression Inventory; CHQ-CF 87 = Child Health Questionnaire-Child Form 87; FAD = Family Assessment Device; FILE = Family Inventory of Life Events; FRI = Family Relationship Index ; HRQOL = health related quality of life; IBD-U = Unspecified IBD; IC = indeterminate colitis; ISSI = Interview Schedule for Social Interaction; LCAI = Lichtiger Colitis Activity Index; PCDAI = Pediatric Crohn's Disease Activity Index; PedsQL = Pediatric Quality of Life Inventory 4.0; PGA = Physician Global Assessment; PIP = Pediatric Inventory for Parents; PUCAI = Pediatric Ulcerative Colitis Activity Index; QoL = quality of life; SAD = Scale of Anxiety in Children; SCL-90 = Symptom Check-List 90; shPCDAI = short Pediatric Crohn's Disease Activity Index; UC = ulcerative colitis; YSR = Youth Self-Report.
